# Acupressure Improves the Weaning Indices of Tidal Volumes and Rapid Shallow Breathing Index in Stable Coma Patients Receiving Mechanical Ventilation: Randomized Controlled Trial

**DOI:** 10.1155/2013/723128

**Published:** 2013-04-23

**Authors:** Suh-Hwa Maa, Chiu-Hua Wang, Kuang-Hung Hsu, Horng-Chyuan Lin, Brian Yee, Karen MacDonald, Ivo Abraham

**Affiliations:** ^1^Department of Somatics and Sports Leisure Industry, College of Humanities, National Taitung University, 369 Shi-Kang Road, Section 2, Taitung 95092, Taiwan; ^2^Graduate Institute of Clinical Medical Sciences, College of Medicine, Chang Gung University, 259 Wen-Hwa 1st Road, Kwei-Shan, Taoyuan 33302, Taiwan; ^3^Laboratory for Epidemiology, Department of Health Care Management and Healthy Aging Research Center, Chang Gung University, 259 Wen-Hwa 1st Road, Kwei-Shan, Taoyuan 33302, Taiwan; ^4^Department of Chinese Medicine, College of Medicine, Chang Gung University, 259 Wen-Hwa 1st Road, Kwei-Shan, Taoyuan 33302, Taiwan; ^5^Department of Thoracic Medicine, Chang Gung Memorial Hospital, No. 5 Fusing Street, Kweishan, Taoyuan 33300, Taiwan; ^6^College of Pharmacy, University of Arizona, 1295 N. Martin, Tucson, AZ 85721, USA; ^7^Matrix45, 6159 W Sunset Road, Tucson, AZ 85743, USA; ^8^Department of Pharmacy Practice and Science, Center for Health Outcomes and PharmacoEconomic Research, College of Pharmacy, University of Arizona, 1295 N. Martin, Tucson, AZ 85721, USA; ^9^Department of Family and Community Medicine, College of Medicine, University of Arizona, 1295 N. Martin, Tucson, AZ 85721, USA

## Abstract

*Background*. Acupressure has been shown to improve respiratory parameters. We investigated the effects of acupressure on weaning indices in stable coma patients receiving mechanical ventilation. *Methods*. Patients were randomly allocated to one of three treatments: standard care with adjunctive acupressure on one (*n* = 32) or two days (*n* = 31) and standard care (*n* = 31). Acupressure in the form of 10 minutes of bilateral stimulation at five acupoints was administered per treatment session. Weaning indices were collected on two days before, right after, and at 0.5 hrs, 1 hr, 1.5 hrs, 2 hrs, 2.5 hrs, 3 hrs, 3.5 hrs, and 4 hrs after the start of treatment. *Results*. There were statistically significant improvements in tidal volumes and index of rapid shallow breathing in the one-day and two-day adjunctive acupressure study arms compared to the standard care arm immediately after acupressure and persisting until 0.5, 1 hr, and 2 hrs after adjustment for covariates. *Conclusions*. In the stable ventilated coma patient, adjunctive acupressure contributes to improvements in tidal volumes and the index of rapid shallow breathing, the two indices most critical for weaning patients from mechanical ventilation. These effects tend to be immediate and likely to be sustained for 1 to 2 hours.

## 1. Introduction

One out of three patients in critical care units requires mechanical ventilation [1, 2]. Invasive mechanical ventilation is potentially harmful due to physical (e.g., gastrointestinal complications [3], pain, and difficult breathing [4]) and psychological factors (e.g., fearfulness and anxiety [4]). Further, 27% of mechanically ventilated patients develop ventilator-associated pneumonia [5] resulting in excess morbidity and mortality. Every effort should be made to wean patients safely from mechanical ventilation within as short a period of time as possible [2].

Acupressure is the massage of acupuncture points [6] performed with the fingertip or knuckle. In noncontrolled studies of patients with respiratory disease, acupressure has been associated with significant subjective improvements in cough [7] and congestion or dyspnea [8, 9] as well as reductions in depression [10] and improvements in quality of life [11], at least for short periods of time. Systematic reviews have argued that more empirical evidence about acupressure is required [12, 13], including evidence of the value of acupressure in the treatment of respiratory disease.

Since acupressure has potential benefits on respiratory symptoms, it may contribute to the success of weaning critically ill patients from mechanical ventilation and hence reduce weaning failure. The purpose of our study was to examine the potential benefits of adjunctive acupressure on objective parameters (weaning indices) of intubated critically ill patients. We hypothesized that stable coma patients with mechanical ventilation randomized to treatment arms receiving standard care and adjunctive acupressure on either one or two days would show improved weaning indices compared to patients receiving standard care only.

## 2. Methods

### 2.1. Design

This study was a prospective randomized clinical trial using a parallel-group design. A sample of medically stable, ventilated coma patients was randomly assigned on a 1 : 1 : 1 basis to one of three treatment groups: two days of standard care with adjunctive acupressure on the first day, two days of standard care with adjunctive acupressure on both days, and standard care on both days. This design enabled us to evaluate the immediacy versus delay of any treatment effects, as well as the duration of these effects above and beyond standard care. This was deemed important because a standardized regimen for delivering acupressure has not been proposed to date. Outcomes were measured before, right after, and at half hour intervals up to 4 hours following acupressure for a total of 10 daily measurements and a trial total of 20 measurements. In the standard care only group, measurements were made at the same time points with a pause inserted where acupressure was administered in the one- and two-day adjuvant treatment arms. 

### 2.2. Participant Selection and Assignment

Participants were recruited from three medical intensive care units (ICU) and one respiratory care center (RCC) unit in a 3715-bed tertiary teaching facility in Taoyuan, Taiwan. Eligibility criteria included being 19 years of age or older, diagnosed with coma and having a Glasgow Coma Scale (GCS) score of less than 7 in the absence of sedation, and having been on mechanical ventilation at 6 to 8 cm H_2_O levels of pressure support preferably but not necessarily between 7 and 21 days. We conservatively selected low levels of ventilator support because, to our knowledge, this was the first study examining the impact of acupressure on weaning indices of intubated patients in the acute stage of mechanical ventilation. Coma patients were chosen because this permitted controlling for nonspecific effects such as positive or negative patient expectations, anxiety about pain, variability in care intensity by the nurse administering acupressure, healing rituals that reference East Asian culture, and interpersonal dynamics in the nurse-patient relationship [14].

Exclusion criteria were as follows: neurologic injury or pathology (e.g., myasthenia gravis and hemiplegia); infection (e.g., open tuberculosis, vancomycin-resistant enterococcus, or pandrug-resistant *Acinetobacter baumannii*); acute cardiac vascular dysfunction; other systemic diseases (e.g., diabetes mellitus, systemic lupus erythematosus, or AIDS); receiving sedation or neuromuscular blockade (e.g., midazolam, barbiturates, opiates, propofol, or pancuronium).

The minimum sample size was determined using the software program Power Analysis and Sample Size (PASS 11.0). In our pilot study of 15 patients, we recorded at baseline and right after acupressure treatment mean tidal volumes of 351.00 ± 104.99 and 407.60 ± 110.92, respectively, and mean indices of rapid shallow breathing of 88.19 ± 44.01 and 70.79 ± 35.56, respectively. Using these estimates, a sample size of 30 patients in each group would be required to detect an effect size of 0.5 with power of 0.80 at *α* = 0.05. Allowing for a 10% loss rate, a total of 99 recruits (33 per group) would be needed.

**Figure 1:**
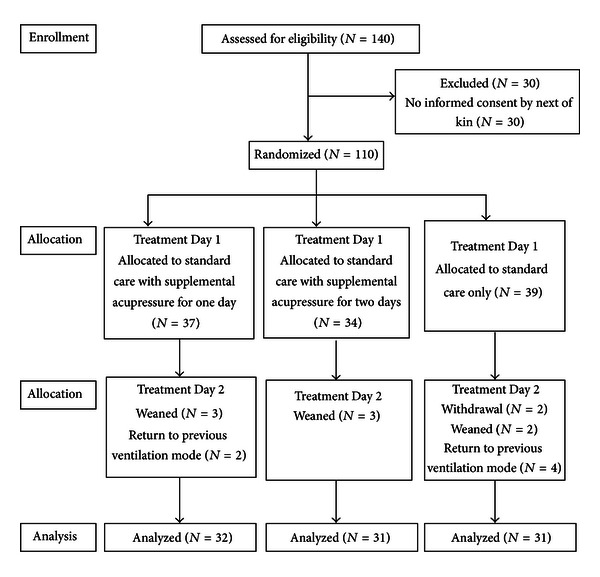
Summary data for study recruitment and completion at each time point: baseline, Day 1 and Day 2.

One-hundred-forty patients met study criteria over a 15-month recruitment period. The next of kin of 110 subjects provided written informed consent (Figure 1). Subjects were randomized to one of the three study arms. The random allocation sequence was concealed until interventions were assigned. Ninety-four patients completed the study protocol. Sixteen patients were withdrawn from the study: consent was revoked for two patients, eight patients were weaned between randomization and protocol completion, and six patients returned to their previous ventilation mode, that is, no longer tolerating pressure support.

### 2.3. Rationale for the Selection of Acupoints

Acupuncture is based on the concept that an energy flow (Qi) essential for good health flows throughout the body along 12 main pathways known as meridians [15]. The patterns of this Qi are related to the organs and the tendon-muscular system. The causes of most disorders in the human body are believed to be due to an imbalance in the Qi [16]. Acupuncture and associated methods such as acupressure may correct the imbalance in the Qi at the related acupuncture points located close to the skin along the meridians. The underlying mechanism of most respiratory disorders is believed to be the result of a defective interaction between lungs and spleen and is explained in great detail (and beyond the scope of this paper) elsewhere [17]. 

**Figure 2:**
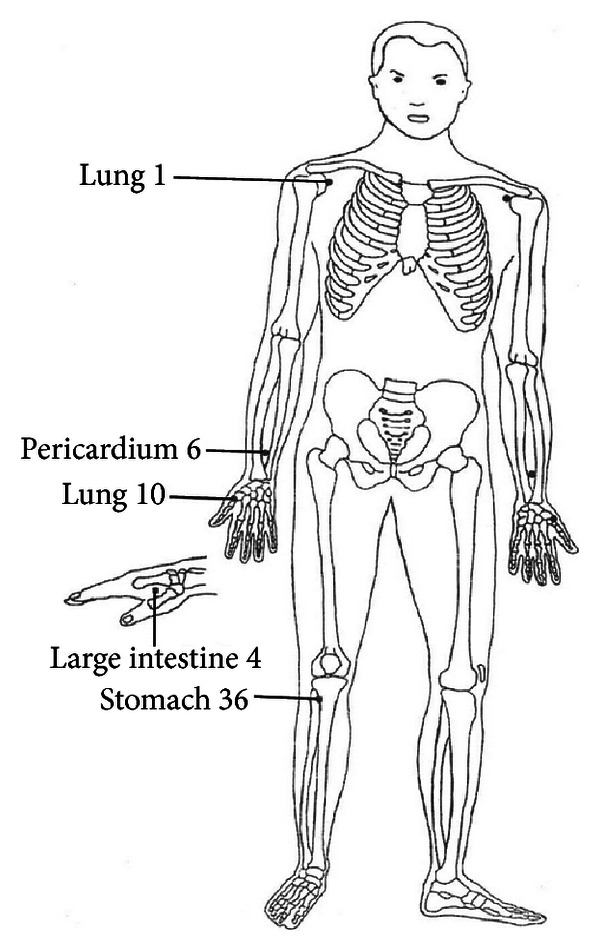
Location of acupressure points. Acupressure points Zhongfu (lung 1, LU1), Yuji (lung 10, LU10), Hegu (large intestine 4, LI4), Neiguan (pericardium 6, PC6), and Zusanli (stomach 36, ST36). Each point is located bilaterally.

The acupuncture points referred to in the present study (Figure 2) were taken from Zhen Jiu Da Cheng (Compendium of Acupuncture and Moxibustion, A.D. 1601), a collection of acupuncture and moxibustion papers from the Han to the Ming dynasties, and edited in 1601 by Yang, a Ming Dynasty acupuncturist [18]. The acupoints selected in this study have been reported to provide substantial relief to patients suffering from dyspnea and to enhance the function of the immune system [16]. The principles of treatment of this study are to relieve any disharmonies of the lung involving the Zhongfu (LU1), Yuji (LU10), Hegu (LI4), and the Neiguan (PC6)) and to strengthen the spleen (Neiguan (PC6) and the Zusanli (ST36)).

According to Stux and Pomeranz [16], the Zhongfu (LU1) is the trigger point (front Mu point) of the lungs as well as the meeting point (Hui Shu point) of lungs and spleen. Manipulating this point oxygenates the lungs, provides relief of symptoms due to asthma, improves the function of the spleen, and relieves both shoulder and back pain. The second point, the Yuji (LU10), is the slow-flowing Ying point and tends to accelerate the flow along the lung meridian. Manipulation of this point relieves pain, cough, and symptoms of asthma. The third point, the Hegu (LI4), is the source point (Yuan point) and has the energy of the combined channel, since the transverse Luo vessel from the Luo point of the combined channel ends at this point. When this point is manipulated, it relieves headache, dizziness, fever, chills, and coughing. The fourth point, the Neiguan (PC6), is the connecting (Luo) point of the pericardium channel to the Yangchi (TE4). It is the point of confluence of the additional channel Yinwei. By manipulating this point the lungs are warmed, sputum is reduced, and the function of the spleen is improved. And finally, the uniting point (He point) is located at the Zusanli (ST36), providing an internal equilibrium for the stomach meridian. The manipulation of this point improves the functioning of the spleen and warms the spleen's yang because it is so closely related to the stomach [16]. 

### 2.4. Treatment Protocols

#### 2.4.1. Standard Care

Throughout the trial all subjects received their prescribed medication and chest physiotherapy, including chest percussion, positioning, and suction.

#### 2.4.2. Adjunctive Acupressure

Acupressure was administered at the five acupoints referenced above. To make sure that appropriate and standard methods were used, acupressure was administered by one of the coinvestigators (C.H.W.), a nurse who had completed three courses of Traditional Chinese Nursing recommended by the Committee of Chinese Medicine and Pharmacy, Department of Health, Executive Yuan (Acupuncture and Moxibustion in Nursing, Traditional Chinese Traumatology in Nursing, and Traditional Chinese Nursing). She also trained clinically in acupressure technique (including intensity, duration, location, and stimulation of the acupuncture points) in the Department of Acupuncture and Moxibustion, Chang Gung Memorial Hospital.

We identified the acupoints by bone standard, which is the length of equally divided portions of a certain long bone or the distance between two anatomical landmarks, taken as one cun, as the unit of measurement for locating acupoints. The stimulation of the five acupoints was done once daily, first to one side of the body and then on the other side, in the order preferred by the patient, by using the fingers to apply gentle but firm pressure [19]. The amount of pressure applied was dependent upon the acupoints location, the skin thickness, and the adipose tissue in that location. The finger was moved in small circles, or in a back and forth motion, but remained located over the same point on the skin, for up to two minutes. Thus acupressure was performed at five acupuncture points for a period of up to 10 minutes per treatment session [7]. Acupressure was performed at the same time, either morning or afternoon, for each participant. If there were two participants recruited at one day, we arranged them to receive acupressure at different times. We did not recruit more than two participants in any one day.

### 2.5. Outcome Measures

Eight outcome measurements (heart rate, respiratory rate, mean arterial pressure, peripheral oxygen saturation [SpO_2_], tidal volumes [*V*_*T*_], minute ventilation [*V*_*E*_], dynamic lung compliance [Cdyn], and rapid shallow breathing index the ratio of the frequency of breathing (*f*) divided by the tidal volume (*V*_*T*_)) were recorded before, right after, and at 0.5 hrs, 1 hr, 1.5 hrs, 2 hrs, 2.5 hrs, 3 hrs, 3.5 hrs, and 4 hrs after the start of treatment, for a total of 10 recordings per day. The rationale for ten daily measurements was that acupressure may be short-acting, hence the frequency of measures at relatively short intervals, and the goal to capture the immediate, lasting, and delayed effects, if any, of acupressure [20].

Heart and respiratory rate, mean arterial pressure, and arterial oxygen saturation scores were obtained from an adequately calibrated and checked electrocardiographic monitor (Hewlett-Packard M1165A Model 56S, Andover, MA, USA). Tidal volume, minute ventilation, and dynamic lung compliance scores were obtained from the median of the five scores from the display on the ventilator, which also was adequately calibrated and checked. 

### 2.6. Statistical Analysis

Multivariate analyses were performed through the use of generalized estimating equations (GEE) of multiple linear regressions with autoregressive 1(AR (1)) correlation [21]. This method takes into account the correlated nature of the data due to repeated measurements after adjusting for the effects of time-dependent and/or time-independent covariates. On the first day a marginal model was used for each dependent variable to establish the population average values of that outcome variable across the ten daily time points after adjusting for the effects of treatment, time, setting, body mass index (BMI), GCS, and baseline values. A similar model was fitted for each dependent variable on the second day. The final model included six covariates: treatment (one-day adjunctive acupressure, two-day adjunctive acupressure, and standard care), time point over two days, corresponding pretreatment (baseline) measurement, setting where patients were treated, BMI, and GCS.

Data were analyzed with SAS v 9.3 (SAS Institute Inc., Cary, NC, USA). The post hoc power analysis was done by using the software program G*Power 3.1.3 [22, 23]. Statistical significance was set at 0.05.

There were no statistically significant differences in baseline characteristics, clinical status variables, and outcome measures between those patients who were withdrawn from the study and those who completed the study protocol. Therefore only the data for those subjects completing the study were analyzed.

### 2.7. Ethical Considerations

The study was approved by the Institutional Ethics Committee of Chang Gung Memorial Hospital. The study was conducted in accordance with the Declaration of Helsinki as subsequently amended. Subjects were enrolled after written informed consent was provided by next of kin.

## 3. Results

### 3.1. Participant Characteristics and Baseline Values

The baseline characteristics of the 94 participants who completed the protocol are shown in Table 1. Mean ± SD age was 73.3 ± 14.3 years. Forty-eight patients were male compared to 46 who were female. Thirty patients were smokers. There were no statistically significant differences between the three groups except that there were, proportionately, more heavy smokers in the one-day acupressure arm and more female subjects in the two-day acupressure arm. Hence we added smoking and gender as confounding variables in the original GEE model. As Table 2 shows, there were no statistically significant differences between the three treatment arms on the outcome variables of interest at baseline.

### 3.2. Outcomes

#### 3.2.1. Tidal Volumes

**Figure 3:**
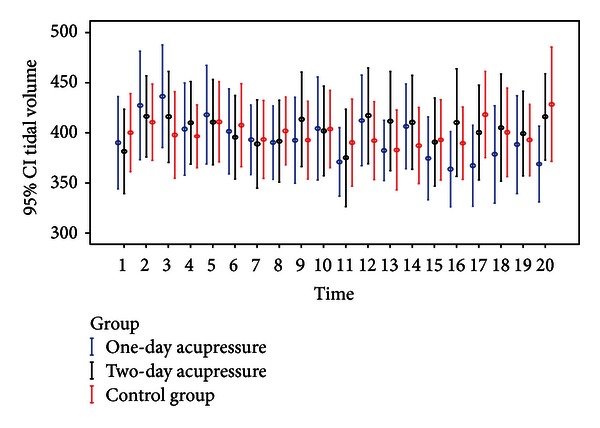
Mean and 95% CI of tidal volume measurement for each treatment arm across the twenty time points.

Tidal volumes (Mean ± SD) observed in the three treatment arms across the 20 time points are presented in Table 3. The 95% confidence intervals (95% CIs) around the means are depicted in Figure 3. The overall mean tidal volumes from baseline to each time point improved after acupressure treatment in both the one-day and two-day acupressure groups on the first day, only in the two-day acupressure group on second day, but not in standard care group. The improvement in tidal volumes following the administration of acupressure was maintained for up to four hours in both acupressure arms on the first day and in the two-day acupressure arm on the second day. No relevant changes were observed in the means of the standard care group.

Statistically significant multivariate analysis results obtained using GEE with AR(1) correlation and controlling for effects of treatment, time, setting, BMI, GCS, and baseline values are reported in Table 5, including estimates of linear regression coefficients, standard errors (SE), and *z* test values and their associated significance levels *P*. On the first day, compared to subjects in the standard care group, change in tidal volumes from baseline was higher by 48.63 mL (*P* = 0.036) at 0.5 hrs for participants receiving one-day adjunctive acupressure. For those in the two-day adjunctive acupressure arm, changes were higher by 32.32 mL (*P* = 0.028) at 1 hr and 39.71 mL (*P* = 0.041) at 3.5 hrs. On the second day, for those in the two-day acupressure arm, changes were higher by 40.10 mL (*P* = 0.008) right after treatment, by 43.35 mL (*P* = 0.014) at 0.5 hrs, and by 38.26 mL (*P* = 0.048) at 1 hr after treatment. There were no statistically significant differences in changes in tidal volumes between the two acupressure groups on either day and across time points (all  *P* = *ns*).

#### 3.2.2. Index of Rapid Shallow Breathing

**Figure 4:**
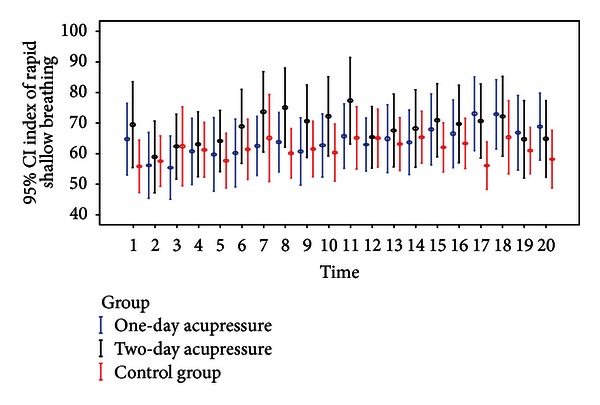
Mean and 95% CI of rapid shallow breathing index for each treatment arm across the twenty time points.

Table 4 lists the mean ± SD values on rapid shallow breathing index across treatment arms and time points, and Figure 4 presents the associated 95% CIs. On the first day, mean scores on rapid shallow breathing index scores decreased after adjunctive acupressure treatment for up to 4 hours in the one-day and for up to 2 hours in the two-day acupressure arms. On the second day, the mean declined for up to four hours in the two-day acupressure group only. No relevant changes were observed in the means of the standard care group.

Multivariate analysis examined changes from baseline in rapid shallow breathing index in subjects in the two adjunctive acupressure arms compared to those in the standard care group after adjustment for covariates (Table 5). On the first day, change from baseline to right after treatment was lower by −10.28 breaths/min/L (*P* = 0.001) in the one-day acupressure arm and by −12.28 breaths/min/L (*P* = 0.003) in the two-day acupressure arm. At 0.5 hrs decreases in the index were −15.77  (*P* = 0.009) and −13.72 breaths/min/L (*P* = 0.029) in the one-day and two-day acupressure groups, respectively. At 1 hr, values were lower by −9.41  (*P* = 0.030) and −11.85 breaths/min/L (*P* = 0.020) in the two respective acupressure groups. The one-day acupressure group also showed a decline by −10.03 breaths/min/L (*P* = 0.039) at 2 hrs. On the second day, subjects in the two-day acupressure arm showed a change of −11.92 breaths/min/L (*P* = 0.001) right after treatment when compared to the standard care group. There were no statistically significant differences in changes in rapid shallow breathing index between the two acupressure groups on either day and across time points.

#### 3.2.3. Other Measures

Scores on the other outcome measures did not show statistically significant changes across the three treatment groups and across time points (data not reported).

#### 3.2.4. Post Hoc Power Analysis

Using the mean ± SD values for tidal volumes (Table 3) and indices of rapid shallow breathing (Table 4), we calculated the corresponding power to ascertain that we had sufficient statistical power. The power estimates at *α* = 0.05 were 0.86 and 0.97, respectively, for the two parameters of interest.

## 4. Discussion

The principal findings of this prospective randomized control trial of the effects of acupressure on weaning indices are two-fold. First, adjunctive acupressure was observed to significantly improve the weaning indices of tidal volumes and rapid shallow breathing index above and beyond the effects of standard care. Second, these effects tended to be immediate and without delayed effect, likely to be sustained for 1 to 2 hours with nominal differentials beyond, and attributable to the acupressure stimulation itself rather than the one- or two-day regimen. Together, these findings suggest that adjunctive acupressure improves the critical weaning indices of tidal volumes and rapid shallow breathing index for a significant but bounded time after treatment. Future randomized controlled trials need to examine whether bilateral stimulation of the Zhongfu (LU1), Yuji (LU10), Hegu (LI4), Neiguan (PC6), and Zusanli (ST36) acupoints, administered for 10 minutes every 1 to 2 hours over sustained periods of time, independently or integrated into weaning protocols, results in accelerated weaning from and decreased rates of relapse to mechanical ventilation.

The differential effect of acupressure on tidal volumes and rapid shallow breathing index but not on six other outcome measures may be attributed to the fact that subjects were clinically stable patients in whom variation in these additional outcome measures is less likely to occur. Further, tidal volumes and rapid shallow breathing index are widely quoted as critical physiological weaning indices [24, 25]. The increased tidal volumes generate adequate transpulmonary pressure gradients to overcome alveolar atelectasis [26, 27]. Decreasing patients' rapid shallow breathing index improves gas exchange and therefore blood gases and blood pH [24].

Although a statistically significant change in tidal volume and rapid shallow breathing index scores was detected in patients receiving acupressure, only those improvements immediately after acupressure exposure as well as 0.5 hr, 1 hr, and 2 hrs after the start of acupressure treatment should be considered clinically significant differences. Additional studies are needed to test the efficacy of different acupressure dosing and timing regimens and to examine the inclusion or exclusion of other acupoints to so optimize acupressure protocols to assist in weaning from mechanical ventilation.

A mechanism of action for the effect of acupressure on tidal volumes and rapid shallow breathing index cannot be proposed at this time. However, a role of hypothalamic and pituitary activation can be hypothesized. Acupuncture (and acupressure by extension) activates myelinated neural fibers that stimulate, among others, the hypothalamus and pituitary gland [28]. This activation releases *β*-endorphins from the hypothalamus into the spinal fluid and the brain and from the pituitary into the blood stream. First, the analgesic effect of *β*-endorphins in general may in itself facilitate respiratory function in patients, improve the effectiveness of breathing movements, and translate into greater tidal volumes. Second and more specifically, the tissue- and muscle-relaxant effect of *β*-endorphins may reduce shallow breathing, enable deeper breathing movements, and thus result in greater tidal volumes. Both may explain why most respiratory patients feel calm, warm, and relaxed during and after acupressure treatments [7, 11], and why most patients with COPD report relief from dyspnea following acupressure [8–10]. Third, deeper breathing movements are associated with increases in plasma *β*-endorphins though it remains unclear whether deeper breathing stimulates *β*-endorphin release or vice versa. In sum, the effect of acupuncture and acupressure on respiratory function may be directly due to the moderating role of these neuropeptides on respiratory function. The effect may also be indirect due to the analgesic and sedative effect of *β*-endorphins, which may facilitate patient breathing.

The use of a randomized controlled design strengthened the internal validity of the study's findings. Being limited to the three intensive care units and one respiratory care center unit of one large medical center limits the external validity and future studies should be multicenter to diffuse any potential class effects for site. Future studies should also examine the effect of acupressure on successful weaning (and relapse as applicable), length of time on mechanical ventilation, length of stay in the critical care unit, and length of hospitalization. Such analyses should be done in general and as a function of the covariates considered in this present study as well as markers of respiratory function, complications of mechanical ventilation, severity of illness, and relevant comorbidities. In addition, about 20–30% of patients do not respond to acupressure regardless of disease condition [29]. Therefore, in order to optimize the efficacy signal, future studies may want to screen patients for prior exposure to acupressure and whether they failed to respond [29]. As noted, optimal acupressure dosing for weaning from mechanical ventilation needs to be established more firmly along with further specification of protocols in terms of clinical administration, intervals between treatments, and overall length of treatment. According to Chinese tradition acupressure should be given repeatedly for sustained improvements [30] but specific recommendations are lacking at this time. Future studies should also broaden the number of respiratory and weaning indices measured and extend beyond stable coma patients. Lastly, future studies should consider including a control group receiving an adjunctive sham treatment. 

## 5. Conclusion

To the best of our knowledge, this trial was the first to investigate the potential benefits of adjunctive acupressure on weaning indices in a group of stable, mechanically ventilated coma patients. The study provides evidence that adjunctive acupressure may improve tidal volume and rapid shallow breathing index scores—the two indices considered most critical to weaning patients from mechanical ventilation.

## Figures and Tables

**Table 1:** Baseline characteristics of participants.

Characteristic	One-day acupressure (*n* = 32)	Two-day acupressure (*n* = 31)	Standard care (*n* = 31)	*P*
	M ± SD	M ± SD	M ± SD	
BMI	22.31 ± 4.78	23.95 ± 5.08	22.83 ± 5.11	0.56*
GCS	4.59 ± 1.50	4.23 ± 1.82	4.81 ± 1.80	0.46*
APACHE II	23.68 ± 8.7^‡^	25.29 ± 7.58^§^	25.42 ± 8.83^||^	0.73*

	*n* (%)	*n* (%)	*n* (%)	

Cigarette pack years				0.38^†^
0	19 (59.4)	24 (77.4)	21 (67.7)	
1–19	2 (6.3)	1 (3.2)	4 (12.9)	
20 or over	11 (34.4)	6 (19.4)	6 (19.4)	
Length of ventilation prior to enrollment, *d*				0.19^†^
≤7	8 (25.0)	12 (38.7)	6 (19.4)	
8 to 13	11 (34.4)	13 (41.9)	10 (32.3)	
14 to 20	8 (25.0)	5 (16.1)	7 (22.6)	
≥21	5 (15.6)	1 (3.2)	8 (25.8)	
Sex, male	20 (62.5)	10 (32.3)	18 (58.1)	0.04^†^
Age, yr, ≥65	28 (87.5)	26 (83.9)	21 (67.7)	0.13^†^
Hypertension history, present	21 (65.6)	17 (54.8)	18 (58.1)	0.68^†^
CVA history, present	14 (43.8)	8 (25.8)	6 (19.4)	0.09^†^
Setting				0.16^†^
MICU-1	3 (9.4)	5 (16.1)	8 (25.8)	
MICU-3	6 (18.8)	11 (35.5)	7 (22.6)	
MICU-5	20 (62.5)	14 (45.2)	11 (35.5)	
RCC	3 (9.4)	1 (3.2)	5 (16.1)	
Intubation				0.27^†^
Endotracheal tube	27 (84.4)	21 (67.7)	25 (80.6)	
Tracheostomy	5 (15.6)	10 (32.3)	6 (19.4)	

Data are presented as mean ± SD or number (%) unless otherwise indicated; *Kruskal Wallis tests; ^†^ *χ* ^2^ and Fisher's exact test; BMI: body mass index; GCS: Glasgow Coma Scale; APACHE: acute physiology and chronic health evaluation. ^‡^ *n* = 25; ^§^ *n* = 21; ^||^ *n* = 24.

**Table 2:** Comparison of baseline measurements among treatment arms.

Variable	One-day acupressure (*n* = 32)	Two-day acupressure (*n* = 31)	Standard care (*n* = 31)	P*
HR, min	91.72 ± 18.10	93.67 ± 18.49	91.58 ± 6.50	0.79
RR, min	21.97 ± 5.39	23.32 ± 7.10	20.74 ± 5.41	0.54
MBP, mm Hg	85.03 ± 15.34	81.81 ± 13.67	83.94 ± 15.44	0.76
SpO_2_, %	96.53 ± 2.21	96.81 ± 2.57	96.45 ± 2.41	0.60
*V*_*T*_, mL	390.00 ± 125.70	381.26 ± 114.17	400.10 ± 105.86	0.83
*V*_*E*_, L/min	8.06 ± 2.34	8.57 ± 3.34	7.83 ± 2.35	0.69
Cdyn, mL/cm H_2_O	34.14 ± 15.88	32.93 ± 13.70	37.81 ± 13.56	0.26
*f*/*V*_*T*_, breaths/min/L	64.74 ± 32.43	69.54 ± 38.10	55.90 ± 23.51	0.40

Data are presented as mean ± SD. *Kruskal Wallis tests; HR: heart rate; RR: respiratory rate; MBP: mean arterial blood pressure; SpO_2_: peripheral oxygen saturation; *V*_*T*_: tidal volume; *V*_*E*_: minute ventilation; Cdyn: dynamic lung compliance; *f*/*V*_*T*_: rapid shallow breathing index.

**Table 3:** Mean ± SD of tidal volume (mL) measurement.

Test time	One-day acupressure (*N* = 32)	Two-day acupressure (*N* = 31)	Standard care (*N* = 31)
0.0 hrs^a^	390.00 ± 125.70	381.25 ± 114.17	400.10 ± 105.86
0.17 hrs^b^	427.21 ± 148.91	416.29 ± 109.62	410.39 ± 103.41
0.5 hrs	436.38 ± 141.72	416.00 ± 122.83	397.84 ± 117.49
1.0 hr	403.34 ± 127.57	409.97 ± 112.23	396.48 ± 85.42
1.5 hrs	417.97 ± 135.84	410.77 ± 115.16	410.87 ± 108.78
2.0 hrs	401.34 ± 116.92	395.61 ± 113.33	407.58 ± 112.27
2.5 hrs	393.06 ± 96.50	389.06 ± 119.77	393.38 ± 105.59
3.0 hrs	390.31 ± 101.53	391.65 ± 110.66	401.74 ± 91.48
3.5 hrs	392.53 ± 118.56	413.48 ± 128.07	392.61 ± 105.75
4.0 hrs	404.28 ± 142.79	401.90 ± 121.61	403.81 ± 104.96
24.0 hrs^c^	370.97 ± 94.65	375.45 ± 131.71	390.10 ± 118.32
24.17 hrs^d^	412.00 ± 124.19	417.03 ± 129.46	391.90 ± 105.09
24.5 hrs	382.50 ± 82.74	411.26 ± 134.14	382.87 ± 108.64
25.0 hrs	406.34 ± 117.17	410.55 ± 127.23	387.26 ± 103.14
25.5 hrs	374.50 ± 114.10	389.68 ± 120.77	393.00 ± 108.86
26.0 hrs	363.90 ± 104.72	410.32 ± 146.53	389.65 ± 98.29
26.5 hrs	367.16 ± 11.75	400.00 ± 128.55	418.23 ± 116.85
27.0 hrs	378.47 ± 134.52	404.81 ± 145.53	400.55 ± 119.9
27.5 hrs	388.25 ± 134.68	399.13 ± 114.63	392.90 ± 96.96
28.0 hrs	369.03 ± 104.09	415.45 ± 116.38	428.48 ± 155.43

^a^Start of treatment on Day 1 (study baseline); ^b^end of treatment on Day 1; ^c^start of treatment on Day 2, if any; also, elapsed time to first Day 2 measurement since study baseline; ^d^end of treatment on Day 2, if any.

**Table 4:** Mean ± SD of rapid shallow breathing index (breaths/min/L) measurement.

Test time	One-day acupressure (*N* = 32)	Two-day acupressure (*N* = 31)	Standard care (*N* = 31)
0.0 hrs^a^	64.74 ± 32.43	69.54 ± 38.10	55.90 ± 23.51
0.17 hrs^b^	56.19 ± 29.77	58.98 ± 31.84	57.61 ± 22.47
0.5 hrs	55.51 ± 28.59	62.35 ± 28.72	62.43 ± 35.07
1.0 hr	60.75 ± 30.11	63.11 ± 28.82	61.32 ± 24.44
1.5 hrs	59.82 ± 33.34	64.15 ± 27.23	57.73 ± 24.28
2.0 hrs	60.28 ± 30.53	68.91 ± 32.85	61.48 ± 26.76
2.5 hrs	62.56 ± 26.85	73.68 ± 35.67	65.17 ± 38.65
3.0 hrs	63.74 ± 26.86	75.06 ± 35.17	60.22 ± 22.05
3.5 hrs	60.80 ± 30.34	70.65 ± 32.24	61.52 ± 24.80
4.0 hrs	62.78 ± 28.24	72.28 ± 35.09	60.41 ± 25.41
24.0 hrs^c^	65.74 ± 29.37	77.26 ± 38.38	65.14 ± 27.81
24.17 hrs^d^	63.02 ± 23.86	65.32 ± 27.14	65.13 ± 25.70
24.5 hrs	64.96 ± 30.71	67.61 ± 32.36	63.20 ± 23.50
25.0 hrs	63.69 ± 29.14	68.21 ± 34.50	65.39 ± 22.76
25.5 hrs	68.03 ± 32.10	70.93 ± 32.45	62.05 ± 21.60
26.0 hrs	66.50 ± 30.77	69.71 ± 34.35	63.31 ± 22.26
26.5 hrs	70.96 ± 27.61	70.72 ± 32.95	56.16 ± 21.00
27.0 hrs	72.88 ± 31.30	72.23 ± 35.58	65.35 ± 32.64
27.5 hrs	66.80 ± 33.66	64.73 ± 34.51	60.98 ± 20.75
28.0 hrs	68.87 ± 30.21	64.84 ± 33.93	58.24 ± 25.60

^a^Start of treatment on Day 1 (study baseline); ^b^end of treatment on Day 1; ^c^start of treatment on Day 2, if any; also, elapsed time to first Day 2 measurement since study baseline; ^d^end of treatment on Day 1, if any.

**Table 5:** Response to treatment over time: covariates effect estimates on tidal volumes and rapid shallow breathing index and standard errors obtained from GEE.

	Estimate	Standard error	*z* value	*P* value
Tidal volumes (mL) at Day 1				
One-day acupressure × 0.5 hrs	48.63	23.20	2.10	0.036*
Two-day acupressure × 1.0 hrs	32.32	14.68	2.20	0.028*
Two-day acupressure × 3.5 hrs	39.71	19.45	2.04	0.041*

Tidal volumes (mL) at Day 2				
Two-day acupressure × 24.17 hrs	40.10	15.16	2.64	0.008*
Two-day acupressure × 24.5 hrs	43.35	17.63	2.46	0.014*
Two-day acupressure × 25.0 hrs	38.26	19.31	1.98	0.048*

The rapid shallow breathing index (breaths/min/L) at Day 1				
One-day acupressure × right after	−10.28	3.20	−3.21	0.001*
Two-day acupressure × right after	−12.28	4.07	−3.01	0.003*
One-day acupressure × 0.5 hrs	−15.77	6.07	−2.60	0.009*
Two-day acupressure × 0.5 hrs	−13.72	6.27	−2.19	0.029*
One-day acupressure × 1.0 hrs	−9.41	4.33	−2.18	0.030*
Two-day acupressure × 1.0 hrs	−11.85	5.09	−2.33	0.020*
One-day acupressure × 2.0 hrs	−10.03	4.86	−2.06	0.039*

The rapid shallow breathing index (breaths/min/L) at Day 2				
Two-day acupressure × 24.17 hrs	−11.92	3.66	−3.26	0.001*

For treatment, the category “control group” was taken as the reference, and either one-day acupressure or two-day acupressure was used as a representative variable for each of the two groups; for time, the category “baseline” at Day 1 was taken as the reference, right after, 0.5 hrs, 1 hr, 1.5 hrs, 2 hrs, 2.5 hrs, 3 hrs, 3.5 hrs, and 4 hrs were representative variables, the category “24 hrs” at Day 2 was taken as the reference, and 24.17 hrs, 24.5 hrs, 25 hrs, 25.5 hrs, 26 hrs, 26.5 hrs, 27 hrs, 27.5 hrs, and 28 hrs were representative variables. For setting, the category “RCC” was taken as the reference. In addition to treatment, time, setting, BMI, GCS, smoking amount, gender, and baseline values were forced into the model to perform GEE. *P < 0.05 compared to controls for the treatment × time interaction.
